# Serum neurofilament light levels correlate with severity measures and neurodegeneration markers in autosomal dominant Alzheimer’s disease

**DOI:** 10.1186/s13195-018-0439-y

**Published:** 2018-11-03

**Authors:** Raquel Sánchez-Valle, Amanda Heslegrave, Martha S. Foiani, Beatriz Bosch, Anna Antonell, Mircea Balasa, Albert Lladó, Henrik Zetterberg, Nick C. Fox

**Affiliations:** 1 Alzheimer’s Disease and Other Cognitive Disorders Unit, Department of Neurology, Hospital Clínic, Institut d’Investigació Biomèdica August Pi i Sunyer, University of Barcelona, Villarroel, 170, 08036 Barcelona, Spain; 2 Department of Molecular Neuroscience, UCL Institute of Neurology, Queen Square, London, UK; 3 UK Dementia Research Institute at UCL, London, UK; 4 Clinical Neurochemistry Laboratory, Sahlgrenska University Hospital, Mölndal, Sweden; 5 Institute of Neuroscience and Physiology, Department of Psychiatry and Neurochemistry, the Sahlgrenska Academy at the University of Gothenburg, Mölndal, Sweden; 6 Dementia Research Centre, University College of London, Institute of Neurology, London, UK

**Keywords:** Alzheimer’s disease, Biomarkers, Familial Alzheimer’s disease, Presenilin 1, Neurofilament light

## Abstract

**Background:**

Biomarkers that can track disease onset and progression in autosomal dominant Alzheimer’s disease (ADAD) are needed. We investigate whether serum neurofilament light (NfL) concentration is associated with clinical and cerebrospinal fluid (CSF) markers in ADAD. We also evaluate serum NfL differences between clinical groups.

**Methods:**

Serum NfL was measured cross-sectionally in 60 individuals from ADAD families using an ultrasensitive immunoassay on the Single molecule array (Simoa) platform and longitudinally in an exploratory study in a subset of six mutation carriers. Spearman coefficients assessed associations between serum NfL and relevant measures. Differences between groups were evaluated by Kruskal-Wallis and Mann-Whitney *U* tests.

**Results:**

Forty-two participants were mutation carriers: 22 symptomatic (SMC) and 20 asymptomatic (AMC). Eighteen subjects were non-carriers and cognitively normal (controls (CTR)). Serum NfL correlated with the estimated years from symptoms onset across mutation carriers (rho = 0.75, *p* < 0.001). In mutation carriers, serum NfL also showed strong correlation with clinical (rho = 0.70, *p* < 0.001) and cognitive (rho = −0.77, *p* < 0.001) measures and CSF NfL, total tau and phosphorylated tau levels (rho = 0.72, 0.71, and 0.71, respectively, all *p* < 0.001). Serum NfL concentration was higher in SMC than in AMC and CTR.

**Conclusions:**

Serum NfL might be a feasible non-invasive biomarker to track disease onset and severity in ADAD.

## Background

Autosomal dominant Alzheimer’s disease (ADAD), with its almost 100% penetrance and relatively predictable age of onset, allows the evaluation of disease-modifying treatments at early or pre-symptomatic stages of the disease [1]. Two trials, the Dominantly Inherited Alzheimer Network Trials Unit and the Alzheimer’s Prevention Initiative [2, 3], are already ongoing. Thus, there is considerable interest in finding non-invasive biomarkers that could track the disease progression or provide evidence of disease modification. Cerebrospinal fluid (CSF) biomarkers have shown strong correlations with clinical and cognitive measures in ADAD [4, 5]. However, repeated CSF sampling is not always feasible or well tolerated. Blood-based biomarkers are less invasive and allow more frequent determinations, although they are challenging due to the lower concentration of brain analytes. Recently, measurement of neurofilament light (NfL) concentration using an ultrasensitive immunoassay on the Single molecule array (Simoa) platform has been demonstrated to be feasible and reliable, both in sporadic Alzheimer’s disease (AD) [6] and ADAD [7], as well as in other neurodegenerative or neuroinflammatory diseases such as frontotemporal dementia [8], progressive supranuclear palsy [9], Huntington’s disease [10], and multiple sclerosis [11].

In this study, we analysed serum NfL levels in a Spanish cohort of ADAD individuals and investigated whether they were associated with clinical markers of disease severity and CSF biomarkers.

## Methods

### Participants

We studied 60 participants from ADAD families caused by 16 different mutations (with number of subjects, both carriers and non-carriers, indicated in brackets): the T116I (*n* = 1), H131R (*n* = 3), M139 T (*n* = 7), H163R (*n* = 2), S169P (*n* = 6), L173F (*n* = 4), G206D (*n* = 2), G209E (*n* = 1), R220G (*n* = 3), L235R (*n* = 3), K239 N (*n* = 8), L282R (*n* = 6), L286P (*n* = 8), G378R (*n* = 2), and I439S (*n* = 2) mutations in the PSEN1 gene and the I716T (*n* = 2) mutation in the APP gene. All the participants were recruited from the genetic counselling programme for familial dementias (PICOGEN) at the Hospital Clinic, Barcelona, Spain [12]. The study was approved by the Hospital Clinic ethics committee and all subjects gave written informed consent.

All participants underwent a complete clinical evaluation, and a comprehensive neuropsychological battery was administered to 52 subjects [12]. Subjects were classified as asymptomatic if they had no cognitive complaints, their cognitive performance was normal, and they scored 0 on the Clinical Dementia Rating (CDR) scale. They were classified as symptomatic if their CDR score was > 0 or if their cognitive performance was ≥ 1.5 standard deviations (SDs) below the mean. We calculated the estimated years from symptom onset (EYO) for asymptomatic mutation carriers (AMC) as the subject’s age at the time of the study minus their parental age at onset. The parental age at onset was determined by a semi-structured interview in which family members were asked about the age of first progressive cognitive decline in the affected parent similar to the Dominant Inherited Alzheimer’s Network [13]. Non-carriers were used as the control population (CTR).

#### Measurement of serum NfL concentrations

The serum NfL concentration was measured using an ultrasensitive immunoassay on the Simoa platform in the DRI Fluid Biomarker Laboratory at UCL London, UK, using the commercially available NF-Light kit according to the manufacturer’s instructions (Quanterix, Lexington, MA). All measurements were performed by specially trained personnel in one round of experiments using one batch of reagents.

#### Measurement of CSF biomarkers

CSF samples were available from 35 participants. Commercially available single-analyte enzyme-linked immunosorbent assay (ELISA) kits were used to determine levels of CSF Aβ1–42, total tau, phosphorylated tau (INNOTEST, Fujirebio-Europe), and NfL (UmanDiagnostics) at the Alzheimer’s Disease and Other Cognitive Disorders Unit Laboratory, Barcelona. This laboratory participates in the Alzheimer’s Association external quality control programme for CSF biomarkers [14].

#### Statistical analysis

We tested the distribution of the values in the sample with the Kolmogorov-Smirnov test. Differences between groups were evaluated by Kruskal-Wallis test and Mann-Whitney test. Spearman correlation coefficients were calculated to assess the association between NfL and EYO and clinical, cognitive, and biochemical measures, first across all participants and all mutation carriers (MC) and then within each group. All statistical analyses were performed using the IBM SPSS (v.20, IBM corp.) software program. Statistical significance was set at *p* < 0.05.

## Results

Twenty-two participants were symptomatic mutation carriers (SMC) and 38 were asymptomatic, with 20 of these being mutation carriers (AMC) and 18 being asymptomatic non-carriers (CTR). AMC were more than a decade younger than their parental age at onset (mean ± SD EYO, −14.26 ± 7.68 years; Table 1). As expected, SMC were significantly older and had lower scores on cognitive and clinical measures. SMC showed higher serum NfL levels compared with AMC and CTR (as well as higher total tau, phosphorylated tau, and NfL, and lower Aβ1–42 CSF levels). No significant differences were observed between AMC and CTR in demographic, clinical, or biochemical variables included in Table 1 except for EYO.

**Table 1:** Participant demographics, cognitive test scores, serum and CSF NfL, and CSF Alzheimer’s disease marker concentrations

	CTR	AMC	SMC	K-W	CTR vs SMC	CTR vs AMC	SMC vs AMC
					M-W U (z; *p*)	M-W U (z; *p*)	M-W U (z; *p*)
*N* = 60 (*N* CSF = 35)	18 (10)	20 (8)	22 (17)				
Age (years), mean (SD)	36.28 (8.51)	34.24 (8.81)	49.16 (10.00)	***p*** **< 0.001**	**57 (–3.83; < 0.001)**	145 (–1.02; 0.38)	**55 (–4.15; < 0.001)**
Sex, M/F	7/10	4/17	9/13	na	na	na	na
EYO (years), mean (SD)	-8.22 (7.9)	-14.26 (7.68)	4.12 (2.52)	***p*** **< 0.001**	**20 (–8.40; < 0.001)**	**106 (–2.16; 0.03)**	**0 (–5.54; < 0.001)**
MMSE, mean (SD)	29.17 (1,04)	29.35 (0,93)	19.14 (5.46)	***p*** **< 0.001**	**3 (–5.34; < 0.001)**	198 (0.58; 0.61)	**2.5 (–5.55; < 0.001)**
CDR, mean (SD)	0	0	1.27 (0.82)	***p*** **< 0.001**	**0 (–5.36; < 0.001)**	na	**0 (–5.36; < 0.001)**
CDR-SOB, mean (SD)	0	0	5.69 (4.15)	***p*** **< 0.001**	**0 (–5.39; < 0.001)**	na	**0 (–5.39; < 0.001)**
Serum NfL (ng/L), mean (SD)	13.85 (5.63)	12.43 (6.48)	30.87 (15.14)	***p*** **< 0.001**	**39 (–4.32; < 0.001)**	142 (–1.11; 0.27)	**39 (–4.56; < 0.001)**
CSF NfL (ng/L), mean (SD)	526.53 (198.89)	517.50 (85.02)	2123.19 (649.45)	***p*** **< 0.001**	**1 (–3.84; < 0.001)**	31 (–0.53; 1)	**0 (–3.60; < 0.001)**
CSF Aβ1–42 (ng/L), mean (SD)	753.98 (231.97)	950.52 (542.67)	327.46 (136.71)	***p*** **< 0.001**	**3 (–4.12, < 0.001)**	49 (0.80; 0.46)	**12 (–3.26; < 0.001)**
CSF total tau (ng/L), mean (SD)	233.70 (73.48)	243.23 (52.12)	1166.60 (958.79)	***p*** **< 0.001**	**2 (–4.17; < 0.001)**	40 (0.00; 1)	**0 (–3.96; < 0.001)**
CSF p-tau (ng/L), mean (SD)	44.63 (10.92)	49.25 (11.00)	153.34 (132.26)	***p*** **< 0.001**	**9 (–3.82; < 0.001)**	50.50 (0.93;0.36)	**12 (–3.26; < 0.001)**

Significant results are indicated in bold typeface

*AMC* asymptomatic mutation carriers, *CTR* non-carriers, CDR Clinical Dementia Rating, CDR-SOB Clinical Dementia Rating sum of boxes, *CSF* cerebrospinal fluid, *EYO* estimated years from symptom onset, *K-W* Kruskal-Wallis test, *M/F* male/female, *MMSE* Mini-Mental State Examination, *M-W U* Mann-Whitney *U* test, *na* not applicable, *NfL* neurofilament light, *p-tau* phosphorylated tau, *SD* standard deviation, *SMC* symptomatic mutation carriers

**Fig. 1:**
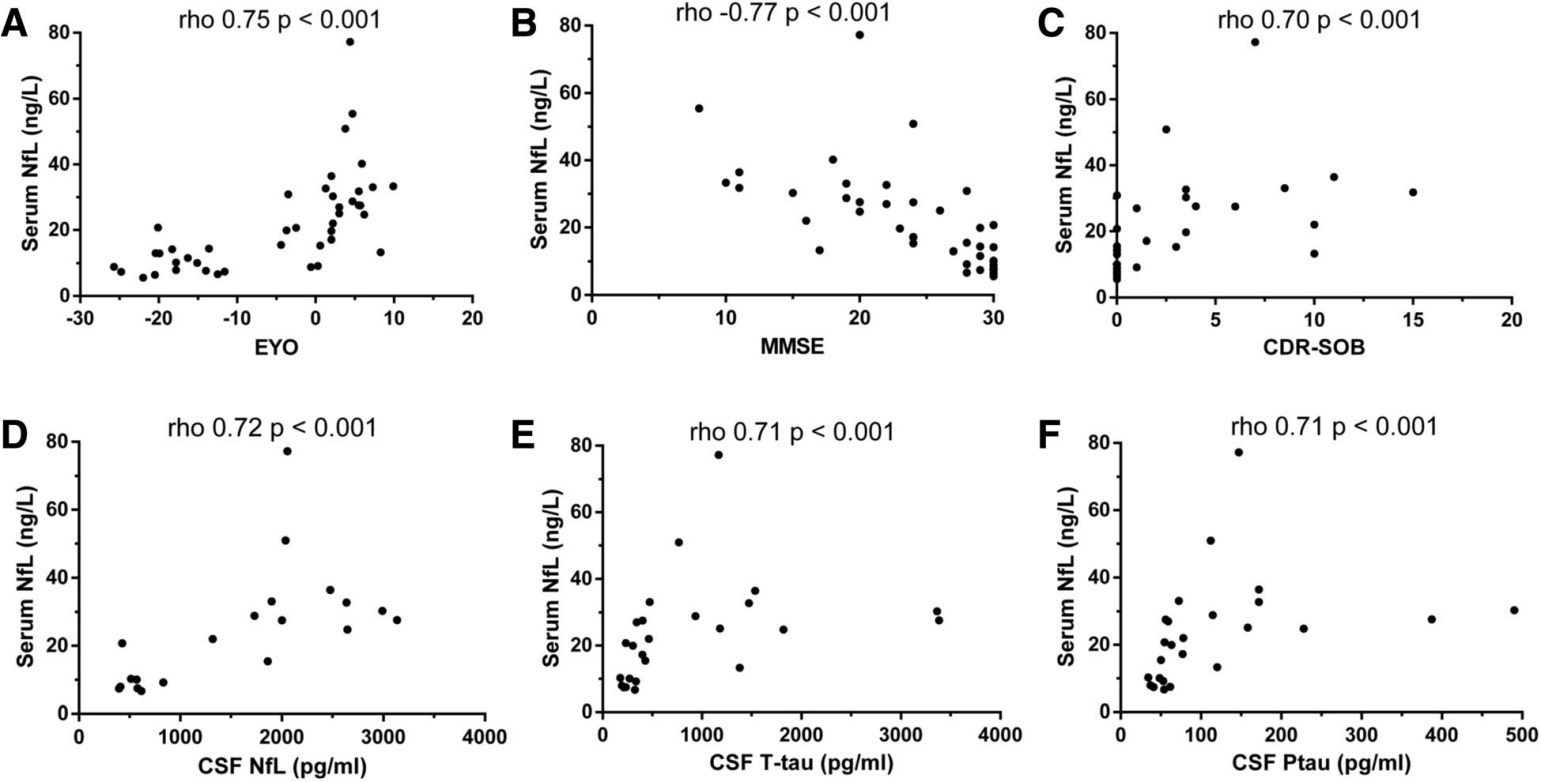
Correlations of serum neurofilament light (NfL) levels with relevant variables in mutation carriers. **a** Estimated years to/from symptom onset (EYO), **b** Mini-Mental State Examination (MMSE), **c** Clinical Dementia Rating sum of boxes (CDR-SOB), **d** cerebrospinal fluid (CSF) NfL, **e** CSF total tau (T-tau), and **f** CSF phosphorylated tau (Ptau)

Serum NfL correlations with age, EYO, and clinical, cognitive, and CSF biochemical measures are shown in Table 2 and Fig. 1. In MC, serum NfL levels showed a negative correlation with Mini-Mental State Examination (MMSE; rho = −0.77, *p* < 0.001) and positive correlations with EYO and CDR sum of boxes (CDR-SOB) (rho = 0.75 and 0.70, respectively, both *p* < 0.001) (Fig. 1a-c and Table 2). When the analysis was restricted to SMC, serum NfL remained inversely correlated with MMSE score (rho = −0.48, *p* = 0.02); when the analysis was restricted to AMC only, we observed a weaker correlation with EYO (rho = 0.41, *p* = 0.073 (two-tailed), *p* = 0.037 (one-tailed)). Serum NfL levels showed significant correlations in MC with several cognitive tests besides MMSE, such as the Free and Cued Selective Reminding test sub-scores: total free recall (rho = −0.54, *p* = 0.02); total recall (rho = −0.68, *p* < 0.001); delayed free recall (rho = −0.57, *p* = 0,01); delayed total recall (rho = −0.57, *p* < 0.001); total Digits score (rho = −0.57, *p* < 0.01); Boston naming test (rho = −0.45, *p* = 0.08); trail making test part A (rho = 0.64, *p* < 0.001), and trail making test part B (rho = 0.60, *p* = 0.002). All these cognitive measures also showed strong significant correlations with MMSE.

**Table 2:** Serum NfL level correlations with demographic, clinical, cognitive, and biochemical measures

	Whole sample rho(*p* value)	CTRrho(*p* value)	MCrho(*p* value)	AMCrho(*p* value)	SMCrho(*p* value)
*N* (*N* CSF)	60 (35)	18 (10)	42 (25)	20 (8)	22 (17)
Age	**0.58 (< 0.001)**	0.03 (0.92)	**0.64 (< 0.001)**	0.35 (0.13)	0.31 (0.16)
EYO	**0.65 (< 0.001)**	−0.25 (0.31)	**0.75 (< 0.001)**	0.41 (0.07)	0.29 (0.19)
MMSE	**−0.65 (< 0.001)**	0.24 (0.34)	**−0.77 (< 0.001)**	−0.30 (0.13)	**−0.48 (0.02)**
CDR-SOB	**0.62 (< 0.001)**	na	**0.70 (< 0.001)**	na	0.35 (0.18)
CSF NfL	**0.70 (< 0.001)**	0.22 (0.58)	**0.72 (< 0.001)**	0.66 (0.16)	0.41 (0.17)
CSF Aβ1–42	**−0.43 (0.01)**	0.04 (0.91)	−0.32 (0.12)	0.24 (0.57)	0.33 (0.19)
CSF total tau	**0.59 (< 0.001)**	**−0.68 (0.03)**	**0.71 (< 0.001)**	0.19 (0.65)	0.39 (0.12)
CSF p-tau	**0.60 (< 0.001)**	−0,56 (0.09)	**0,71 (< 0.001)**	0.19 (0.65)	0.41 (0.10)

Significant results are indicated in bold typeface

*AMC* asymptomatic mutation carriers, *CTR* non-carriers, CDR-SOB Clinical Dementia Rating sum of boxes, *CSF* cerebrospinal fluid, *EYO* estimated years from symptom onset, *MC* mutation carriers, *MMSE* Mini-Mental State Examination, *na* not applicable, *NfL* neurofilament light, *p-tau* phosphorylated tau, *SMC* symptomatic mutation carriers

Serum NfL correlated with CSF NfL (rho = 0.72, *p* < 0.001), total tau (rho = 0.71, *p* < 0.001), and phosphorylated tau (rho = 0.71, *p* < 0.001) (Fig. 1d-f) but not with Aβ1–42 levels (rho = −0.32, *p* = 0.12) in MC. In CTR, serum NfL correlated with CSF total tau levels, but not with other markers.

**Fig. 2:**
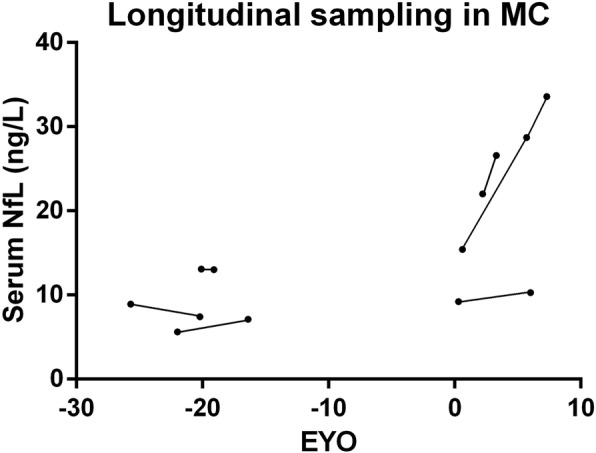
Individual paired (basal + longitudinal) serum neurofilament light (NfL) levels. EYO estimated years to/from symptom onset. MC mutation carriers

Six available longitudinal serum samples from MC obtained at least 1 year apart were also analysed. The mean ± SD annual longitudinal change was 1.67 ± 1.84 ng/L (Fig. 2). This value correlated with the baseline EYO (rho = 0.89, *p* = 0.019). No longitudinal samples from CTR were available for comparison.

## Discussion

We found that serum NfL concentrations correlated with severity measures in ADAD mutation carriers, as well as with CSF NfL, total tau, and phosphorylated tau levels. Serum NfL levels in MC correlated with the estimated years to symptom onset (EYO). In participants who were already symptomatic (SMC), NfL levels correlated with global cognition (MMSE). SMC showed increased NfL levels compared with both CTR and AMC but we did not find a significant difference between the CTR and the AMC groups who were, as a group, 14 years younger than the parental age of onset. In a previous study, Weston and colleagues studied serum NfL with the same methodology in 48 individuals from a different ADAD cohort [7]. In this cohort, like ours, serum NfL correlated with EYO and cognitive measures across MC. Different studies suggest that NfL is a non-specific marker of neurodegeneration [15]. Serum NfL levels are elevated and may reflect disease intensity, not only in sporadic AD [6] but also in amyotrophic lateral sclerosis [16], frontotemporal lobar degeneration [8], Huntington’s disease [10], and Creutzfeldt-Jakob disease [17]. However, more longitudinal data are needed to evaluate the reliability of serum NfL in monitoring the progression of neurodegeneration. Promisingly, serum NfL levels are normalized in response to disease-modifying therapies in multiple sclerosis [18].

NfL may also be useful for predicting symptom onset in genetic neurodegenerative conditions such as frontotemporal dementia [19], genetic Creutzfeldt-Jakob disease [17], or genetic amyotrophic lateral sclerosis [20], although it is still unclear when increased levels can first be detected in these different disorders and whether NfL rises in the asymptomatic phase of each disease or only with symptom onset.

Even if the mean concentrations were similar in the two studies (CTR 12.7 ± 7.2 pg/mL; AMC 16.7 ± 7.7 pg/mL, and SMC 46.0 ± 20.8 pg/mL), Weston and colleagues reported significantly increased serum NfL in AMC compared with CTR in ADAD; that differed from our results. Differences in the mean EYO in AMC could account for this discrepancy, as serum NfL correlated with EYO in both cohorts and the asymptomatic carriers were closer to symptom onset in the UCL cohort than in our study. Similarly, although they should be considered exploratory results due to the limited sample size and the absence of controls, in our study the serum NfL levels increased longitudinally with the rate of change in SMC higher than in the AMC, suggesting that the magnitude of annual NfL change might increase with disease progression. Thus, these results would support the idea of a progressive increase in serum NfL and, thus, neurodegeneration during the asymptomatic phase of the disease that might accelerate around the time of symptom onset. No individuals in the severe phases of the disease were included in any of the studies to evaluate if the increase in serum NfL is maintained in advanced phases of the disease.

Serum NfL levels significantly correlated with CSF NfL levels in the whole cohort and across MC as described in other studies in different neurodegenerative diseases [6, 15, 19]. Serum NfL also correlated with CSF total tau and phosphorylated tau levels, although these correlations were not statistically significant within diagnostic groups, suggesting that the pathological condition reflected by each biomarker may diverge in different stages of the AD continuum, as has been shown in sporadic AD [6]. However, we cannot rule out a type II error (false negative result) in our study due to the small sample size when AMC and SMC were analysed separately.

There are several limitations in this study. Although the sample size is relatively large for the rarity of ADAD, the sample size limited the interpretation of some of the analyses and it would be of interest to explore these results in larger cohorts. It would be of great interest to know if different mutations have a different effect on serum NfL levels, but unfortunately the low numbers of subjects from each mutation preclude this analysis. The lack of repeated individual samples in most of the participants also limits the interpretation of the reliability of the marker at the individual level in longitudinal studies.

## Conclusions

In summary, serum NFL levels in ADAD MC are associated with expected time to symptom onset, with clinical and cognitive measures, and with CSF neurodegeneration markers. These findings suggest that serum NfL may be a non-invasive biomarker for the prediction of symptom onset and potentially for tracking disease severity in ADAD.

## Abbreviations

AD: Alzheimer’s disease
ADAD: Autosomal dominant Alzheimer’s disease
AMC: Asymptomatic mutation carriers
CDR: Clinical Dementia Rating
CDR-SOB: Clinical Dementia Rating, sum of boxes
CSF: Cerebrospinal fluid
CTR: Controls (non-carriers)
EYO: Estimated years from symptom onset
MC: Mutation carriers
MMSE: Mini-Mental State Examination
NfL: Neurofilament light
SD: Standard deviation
SMC: Symptomatic mutation carriers
